# Biomimetic Precapillary Flow Patterns for Enhancing Blood Plasma Separation: A Preliminary Study

**DOI:** 10.3390/s16091543

**Published:** 2016-09-21

**Authors:** Bumseok Namgung, Justin Kok Soon Tan, Peter Agustinus Wong, Sung-Yong Park, Hwa Liang Leo, Sangho Kim

**Affiliations:** 1 Department of Biomedical Engineering, National University of Singapore, Singapore 117583, Singapore; cienab@nus.edu.sg (B.N.); bietksj@nus.edu.sg (J.K.S.T.); peter.wong@u.nus.edu (P.A.W.); bielhl@nus.edu.sg (H.L.L.); 2 Department of Mechanical Engineering, National University of Singapore, Singapore 117575, Singapore; mpeps@nus.edu.sg

**Keywords:** microfluidics, cell-free layer, hydrodynamic separation, hemodynamics

## Abstract

In this study, a biomimetic microfluidic plasma separation device is discussed. The design of the device drew inspiration from in vivo observations of enhanced cell-free layer (CFL) formation downstream of vascular bifurcations. The working principle for the plasma separation was based on the plasma skimming effect in an arteriolar bifurcation, which is modulated by CFL formation. The enhancement of the CFL width was achieved by a local hematocrit reduction near the collection channel by creating an uneven hematocrit distribution at the bifurcation of the channel. The device demonstrated a high purity of separation (~99.9%) at physiological levels of hematocrit (~40%).

## 1. Introduction

Since the turn of the century, microfluidic-based devices have garnered great interest in medical diagnostics. Many blood analyses require the separation of plasma from whole blood due to the rich protein content in blood plasma. This, however, demands a high separation purity and volume of separated plasma for better diagnostic outcomes. Plasma separation is traditionally performed by centrifugation, which is perceived as the gold standard. However, the process of centrifugation is time-consuming and requires a trained operator in a dedicated laboratory space. The process is also non-standardized and carries the risk of sample contamination [1]. Moreover, a high centrifugal force could potentially result in shear-induced red blood cell (RBC) lysis (hemolysis), which in turn leads to platelet and white cell activation. Consequentially, these potential limitations significantly decrease reproducibility and accuracy of the analyses.

To overcome these limitations, a number of microfluidic devices have been proposed based on different separating mechanisms, such as field flow fractionation, filtration, and hydrodynamic separation [2,3,4,5,6,7,8,9,10,11,12,13]. Of these, passive microfluidic devices (devices that rely solely on hydrodynamic or geometry-induced phenomena) offer advantages such as speed and ease of use, while negating the need for any sophisticated external devices or complicated setups. Despite these advantages, some limitations still exist for passive devices. Most of these devices have complicated designs and complex 3D geometries that may limit parallelization of multiple channels to enhance the throughput and integration as an add-on module. 

Several of the previous passive devices exploited the concept of cell-depletion region or cell-free layer (CFL) formation and its consequential influence on plasma skimming in micro-blood flow for plasma extraction [4,5,6,14]. CFL formation is an important hemodynamic feature that can be found in microvascular blood flow [15]. The CFL formation adjacent to the wall of the collection branch side promotes plasma skimming, in which the CFL becomes a barrier to transverse migration of RBCs crossing the streamline toward the collection branch. Consequentially, the increase in the CFL width before the bifurcation can enhance the plasma skimming and reduce the tendency of RBCs to enter the collection branch. To this end, many plasma skimming devices have focused on condensing the RBCs to a particular region and enhancing the CFL formation [4,6]. To achieve this, geometric features such as cavities, sudden expansions, and contractions [4] have been introduced to enhance the CFL width. 

Our previous in vivo studies [16,17,18] have demonstrated that the CFL width is influenced by topological factors and local flow conditions. Thus, the present study proposes a biologically inspired microfluidic chip for plasma separation by drawing on in vivo observations from arteriolar bifurcation flows. The Zweifach-Fung bifurcation law is exploited to artificially modulate the channel hematocrit in the daughter channels to increase separation purity. The device performance was evaluated while varying a design parameter and flow rate.

## 2. Materials and Methods

### 2.1. Biomimetic Approach from in Vivo Observations

**Figure 1:**
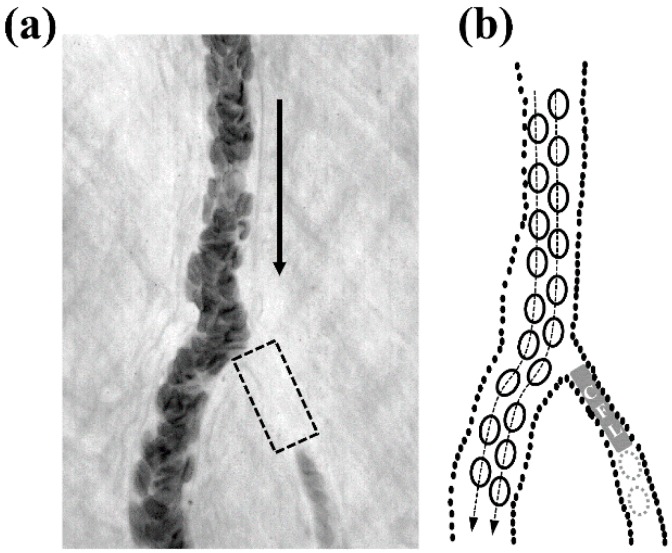
In vivo observation of plasma skimming at an arteriolar bifurcation (**a**); And its corresponding schematic depicting the red blood cell (RBCs) flow trajectory (**b**); Dashed and solid arrows indicate the cell trajectory and flow direction, respectively. Dashed cells indicate an absence of RBCs in the daughter branch. Plasma from the cell-free layer (CFL) can be observed to skim off into the daughter branch.

In vivo flow images were obtained from arteriolar bifurcation flows in the rat cremaster muscle as described in our previous studies [16,17,18]. Figure 1 shows a typical example of plasma skimming in an arteriolar bifurcation (internal diameter (*ID*_parent_) = ~37 μm, mean arterial pressure (MAP) = 86 mmHg) where the upstream vessel and daughter branches were denoted by ‘parent’ and ‘daughter’, respectively. Pure plasma flow was observed in the smaller daughter vessel (highlighted with a gray rectangular box in Figure 1 due to plasma skimming, which was induced by the formation of the CFL before the bifurcation point. In addition, the higher flow in the main daughter vessel was more favorable to attract RBCs flow. Accordingly, the lower flow rate vessel (the right daughter vessel in Figure 1) saw a lower incidence of RBCs flow. 

**Figure 2:**
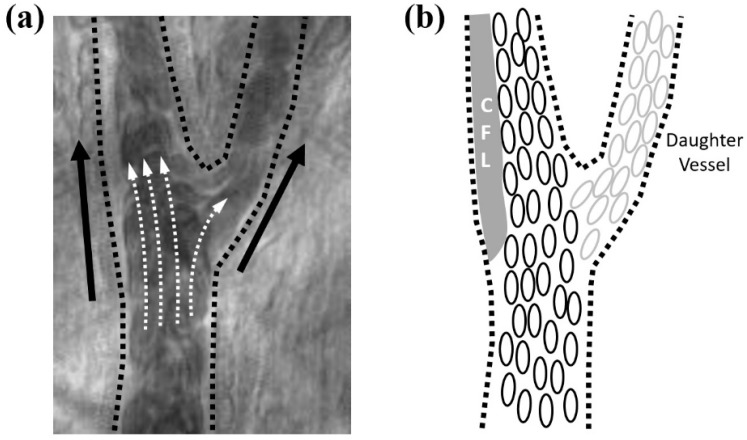
In vivo observation of CFL enhancement due to a local hematocrit reduction at an arteriolar bifurcation (**a**) and its corresponding illustration for RBCs trajectory (**b**); Dashed and solid arrows indicate the cell trajectory and flow direction, respectively. Bold cells represent RBCs of higher concentration in the main channel while gray cells indicate a lower concentration of RBCs in the daughter vessel.

Figure 2 depicts a typical example of CFL enhancement stemming from a local hematocrit reduction near an arteriolar bifurcation (*ID*_parent_ = 47 μm, MAP = 101 mmHg). The wider CFL was formed adjacent to the vessel wall opposite to the side of daughter vessel. A great difference in flow rate ratio between parent and daughter vessels induced the prominent CFL formation by reducing the local hematocrit in the parent vessel after the bifurcation. This is further enhanced by the inertial effect of flowing RBCs in the parent vessel. 

### 2.2. Microchannel Design and Principle

**Figure 3:**
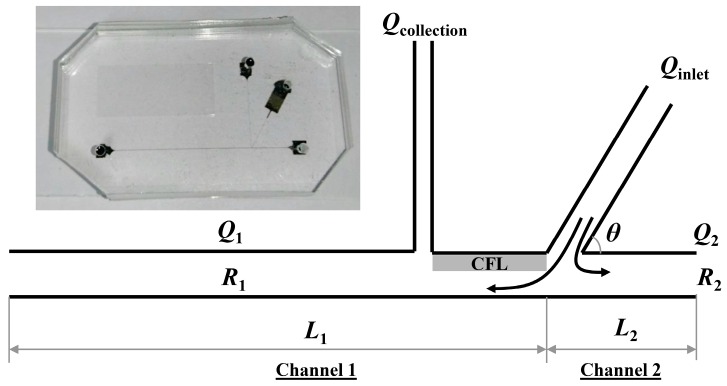
Schematic diagram of the microchannel design for plasma separation. The gray area indicates the region where CFL width measurements were performed. Due to the difference in flow resistance in channels 1 and 2, RBC flow was biased towards the channel with the higher flow rate, enhancing the CFL formation in the collection channel. The inset shows a photograph of the fabricated microchannel.

A microchannel was designed based on in vivo arteriolar bifurcations (Figure 1 and Figure 2). A simple Y-shape bifurcation channel was considered for collecting plasma from whole blood by enhancing the plasma skimming effect (Figure 3). The microchannel comprised a flow inlet, which bifurcated into two daughter channels (channels 1 and 2). Channel 1 featured an additional collection channel downstream of the bifurcation. The enhancement of the effect was achieved by maximizing the CFL width adjacent to the plasma collection channel (*Q*_collection_). To maximize the CFL width, we varied the flow resistance ratio between the two outlets (*R*_1_:*R*_2_ where the subscripts 1 and 2 refer to channels 1 and 2, respectively), which resulted in the biased local hematocrit in channel 2 as compared to channel 1. This, in turn, culminated in a significant reduction in local hematocrit in channel 1. Since the RBCs have a tendency to flow into a branch with a higher flow rate (*Q*_2_), the high flow channel would have a higher cell concentration. On the other hand, channel 1, having a lower flow rate (*Q*_1_), will have a lower cell concentration based on the Zweifach-Fung effect [19,20].

The local hematocrit reduction for enhancement of the CFL width near the plasma collection channel (*Q*_collection_) was achieved by varying the flow rate ratio between the two downstream branches (*Q*_1_:*Q*_2_ = 1:4). The flow rate ratio was determined by resistance difference (*R*_1_:*R*_2_ = 4:1) resulting from the channel length ratio between the two outlet branches (*L*_1_:*L*_2_ = 4:1). The lengths of channel 1, channel 2, the collection channel, as well as the distance between the inlet and collection channel were 2 cm, 0.5 cm, 0.5 cm and 60 µm, respectively. Various ranges of inlet flow rates (*Q*_inlet_: 20, 40, 80 µL/min) and angles (θ = 30°, 45°, 60°) were examined for maximizing the plasma separation. The channel widths of the inlet, outlet and collection channels were 15, 30, 5 µm respectively. The depth of the channels was 30 μm. 

All the microfluidic channels were fabricated with polydimethylsiloxane (Sylgard 184 PDMS; Dow Corning, MI, USA) based on the standard photolithography and soft lithographic procedures. SU-8 2075 negative photoresist (MicroChem, Westborough, MA, USA) was first spin-coated onto a polished silicon wafer to achieve the desired thickness. The mold was subsequently subjected to soft baking, UV exposure, and post baking. SU-8 developer (MicroChem, Westborough, MA, USA) was then used to develop the features on the mold. To fabricate the microchannels, PDMS prepolymer and curing agent were mixed in a 10:1 ratio (w/w) and poured onto the silicon mold before degassing and baking. The PDMS microchannel was then peeled off from the mold, and inlet and outlet ports were punched using a 1.5 mm biopsy punch. The microchannel was then irreversibly bonded to a microscope glass slide by oxygen plasma treatment. To prevent potential RBC adhesion to the channel wall, the channels were pre-perfused with a 1% bovine serum albumin (BSA, Sigma-Aldrich, St. Louis, MO, USA) solution.

### 2.3. Blood Sample Preparation

Human blood containing 7.5% K_2_EDTA (I-DNA Biotechnology, Singapore) was centrifuged (Sigma 2-6, Sigma Laborzentrifugen GmbH, Osterode am Harz, Germany) and washed with 1X Phosphate Buffer Saline (PBS, pH 7.4). The buffy coat was gently removed after centrifugation and the blood sample was washed three times more before collecting packed RBCs. Hematocrit of the blood samples was adjusted to be 40% in the PBS suspending medium and it was verified by a microhematocrit centrifuge (Sigma 1-14 Microcentrifuge, Sigma Laborzentrifugen GmbH, Osterode am Harz, Germany). The rationale for washing the blood sample was to exclude the effect of varying hematocrit on the separation performance with varying parameters (flow rate and bifurcation angle). However, we verified that the use of whole blood displayed no significant difference (in terms of the normalized CFL width) from washed RBCs resuspended at a matched hematocrit (Supplementary Figure S1). 

### 2.4. Cell-Free Layer Width Measurement

The channel flow was observed on an inverted microscopic stage (IX71, Olympus, Tokyo, Japan) with a 40X objective (UPlanSApo 40x, Olympus, Tokyo, Japan) and a long working distance condenser (WI-UCD, Olympus, Tokyo, Japan). The flow was recorded at 500 frame/s with a monochrome high-speed video camera (FASTCOM-1024PCI, Photron, San Diego, CA, USA) for 10 s. A blue filter (model NO. B390, HOYA, Tokyo, Japan) with peak transmission at a wavelength of 394 nm and spectral bandpass at 310–510 nm was used to enhance the contrast between RBCs and the background. The inlet flow rate (*Q*_inlet_) was controlled by a syringe pump (KDS 210, KD Scientific, Inc., Holliston, MA, USA).

The working principle of our device relies on the enhancement of plasma skimming by increasing the CFL width near the plasma collection channel (Figure 3). Accordingly, evaluation of our device was performed by measuring the CFL width. The CFL width is defined by the distance from outermost edge of the RBC core to the inner channel wall. The detailed description of the CFL width measurement can be found in our previous study [21]. The spatial resolution of the CFL width measurement in the present study was ~0.5 μm. The CFL width measurement was performed downstream along the upper wall between *Q*_collection_ and *Q*_inlet_ since the CFL formation before the plasma collection would be critical for the separation (Figure 3). 

### 2.5. Statistical Analysis

All statistical comparisons were performed with a statistical software package (Prism 6.0, GraphPad Software, Inc., La Jolla, CA, USA). For all statistical tests, *P* < 0.05 was considered to be statistically significant. All the experimental results in the present study were represented as mean ± SEM from five repetitive experiments for each condition.

## 3. Results and Discussion

**Figure 4:**
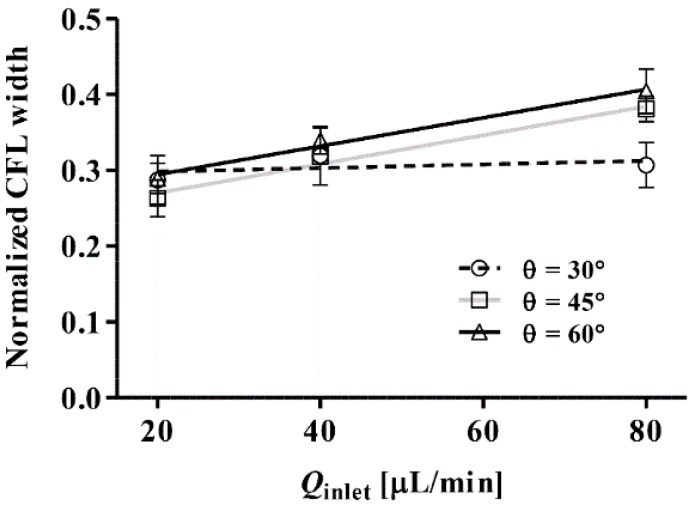
Effect of inlet flow rate (*Q*_inlet_) on the normalized CFL width with different angles (θ). The lines represent linear regression fits for each data set.

Figure 4 shows the effect of inlet flow rate (*Q*_inlet_) on the normalized CFL width for different inlet angles (θ). The CFL width was measured near the collection channel, and it was normalized by the downstream channel width (30 μm). A significant linear relation between the CFL width and *Q*_inlet_ was found for θ = 45° and 60° (*P* < 0.01). The CFL width exhibited an apparent independence from *Q*_inlet_ when θ = 30°. This may be attributed to the dominance of the horizontal component of the RBC momentum over the vertical component at such an acute angle. As θ increased to 45° and 60°, RBCs entering from the inlet channel were pushed closer to the bottom wall of the collection channel. It is of note that we did not perform experiments for θ = 90° since it was thought that the CFL width, and hence the plasma yield and purity, would be reduced as the bifurcation angle increases to 90°. This may be understood by considering the effect of the bifurcation angle on the pressure loss at the bifurcation point. At the bifurcation angle increases, the pressure loss due to viscous losses (as the flow changes direction) reduces the flow velocity in the daughter channel 1. As such, the pressure driving the flow in channel 1 is reduced. Hence, in accordance with the Zweifach-Fung Bifurcation Law, the difference in pressure between channel 1 and the collection channel is reduced, hence reducing the thickness of the CFL.

**Figure 5:**
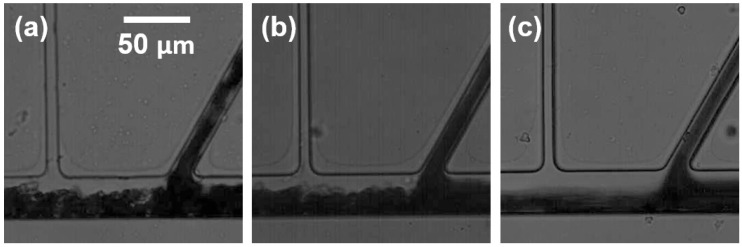
Typical examples for plasma separation from 40% hematocrit blood samples with the bifurcation angle of θ = 60° at 20 µL/min (**a**); 40 µL/min (**b**) and 80 µL/min (**c**) of flow rate (*Q*_inlet_).

Figure 5 shows typical examples of plasma separation from a blood sample (40% hematocrit) at a fixed angle (θ = 60°). The images clearly illustrate the prominent CFL formation near the side of the collection channel regardless of inlet flow rates (*Q*_inlet_). An asymmetric CFL formation between the left and right outlet channels was observed due to the flow rate difference induced by the resistance ratio in the outlet channels. The volume fraction of RBCs in a bifurcation has been previously explained by the Zweifach-Fung effect [19,20] in which RBCs preferentially flow into a higher flow rate vessel than the other. Due to the particulate nature of blood flow, the mass flux fraction deviated from the volume flow fraction while increasing the flow rate, owing to the inertia of the RBCs. This resulted in a large asymmetric hematocrit redistribution, which consequently reduced the local hematocrit in the side of the collection channel. Correspondingly, the CFL width was considerably larger in the low flow rate channel. The hematocrit difference in the outlet channels is due to the resistance difference, thus the CFL is expected to increase with increasing resistance difference (Supplementary Figure S2).

The CFL width near the collection channel was widened with increasing *Q*_inlet_. The Reynolds number (Re= ρvd/μ; ρ is the blood density, v is the mean flow velocity, d is the vessel diameter, and μ is the blood viscosity) in the inlet flow for each flow condition was 2.96, 5.93, and 11.9 for 20, 40, and 80 μL/min, respectively. This increase in the CFL width with increasing *Q*_inlet_ could be due partly to the enhanced inertial effect of RBCs flowing out of the inlet channel. Under higher inertial conditions, more RBCs could be pushed towards the bottom wall of the downstream channel (Figure 5c), consequentially manifesting in a thicker CFL near the upper wall of the channel. 

**Figure 6:**
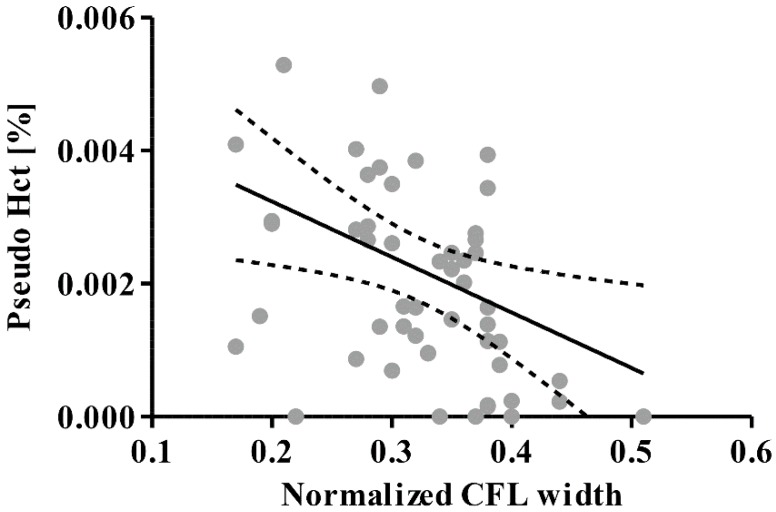
Pseudo hematocrit (Hct) of separated plasma as a function of CFL width. Solid and dashed lines represent a regression fit and 95% confidence band, respectively.

To evaluate the efficacy of the plasma separation, the pseudo hematocrit was obtained (Figure 6). The pseudo hematocrit was defined by the total cell volume over total volume of collected plasma during a given time period (10 s). The total cell volume was measured by counting the number of cells which entered the collection channel over the 10 s period. The RBC volume was assumed to be 100 fL based on previously reported literature [22]. Thus, lower values than unity indicate lower purity of collected plasma and the zero value represents 100% purity. The range of pseudo hematocrits obtained regardless of measured CFL widths indicated a plasma purity of approximately 99.9%. This high purity evidently proves that our device is comparable to currently available devices. However, it should be noted that the purity levels reported in most previous studies were obtained with diluted blood samples at hematocrit levels from 5% to 30% [4,5]. Yang et al. [5] achieved a 99.98% plasma selectivity with 35% of sheep blood at a very low inlet flow rate of 10 µL/h. Sollier et al. [4] managed to extract plasma at a purity of close to 100% at an inlet flow rate of 50 µL/min with 5% hematocrit. The maximum shear stress exerted on the RBCs at the highest flow rate was approximated to be 15 Pa, which is much less than the threshold shear stress of 150 Pa reported in a previous study [23] for shear-induced RBC lysis. As such, the collected plasma was assumed to be devoid of contamination by products of RBC lysis, which can compromise the quality of the collected plasma. We demonstrated a simple method of obtaining highly pure plasma from whole blood, without any complex channel geometries or system configuration. Multiple channels may be connected in series to enhance the recovery of the plasma, and the simplicity of the operation suggests the potential for the device to be incorporated into point-of-care (POC) devices for disease diagnostics. 

Table 1 provides a summary of the recent plasma separation techniques, as well as the corresponding metrics for comparison between the different platforms. Here, the purity is defined by 1-(C_out_/C_in_) where C_out_ is the number of RBCs in the collected plasma and C_in_ is the number of RBCs in the sample inlet. The separation efficiency indicates the volume fraction of the collected plasma over the total plasma volume of the sample. Compared to the previous platforms which separated plasma based on the plasma skimming effect, the current device is able to process a higher concentration of blood (whole blood) and still achieve 100% purity of the collected plasma. Although the flow velocity is considerably lower than other platforms, this ensures that the shear rate is sufficiently low to prevent RBC lysis. As seen in the table, previous studies have also introduced other microfluidics-based platforms for plasma separation; these include active methods such as centrifugation force techniques, acoustophoresis and dielectrophoresis. The benefit of our proposed method is the simplicity of the design, which allows for easy parallelization for increased throughput and ease of integration with other downstream analysis components.

## 4. Conclusions

In this study, we introduced a simple but effective way of enhancing the plasma separation from blood based on an in vivo bifurcation flow phenomenon. The uncomplicated design allows for ease of fabrication and parallelization. The variation of inlet flow rate and bifurcation angle could modulate the throughput of the separation. Our results demonstrated that a larger bifurcation angle (θ = 60°) with a higher flow rate (80 μL/min) would provide a higher separation purity by enhancing the CFL width in the low flow rate channel, adjacent to the collection channel. The biased local hematocrit distribution in the flow splitting zone resulted in a great reduction of local hematocrit near the collection side, which, in turn, significantly decreased the possibility of RBCs flowing into the collection channel, hence increasing the purity of collected plasma.

## Figures and Tables

**Table 1:** Plasma separation techniques.

Authors	Principle	Purity (%)	Separation Efficiency (%)	Velocity (µL/min)	Hematocrit (%)
Prabhakar et al. [12]	Zweifach-Fung, plasma skimming, Centrifugal force	80	-	600	Whole blood
Marchalot et al. [24]	Cell free recirculation zones	-	17	175	2
Tripathi et al. [25]	Zweifach-Fung, plasma skimming	99.7	1.81	150	2
Fekete et al. [26]	Zweifach-Fung	68	12.5	30	1
Zhang et al. [27]	Delamination and sedimentation	99	66	15	8
Kersaudy-Kerhoas et al. [14]	Zweifach-Fung and Cell Free Layer	100	5	33.3	30
Kersaudy-Kerhoas et al. [28]	Zweifach-Fung	53	40	167	3
Rodrigues-Villareal et al. [3]	Zweifach-Fung, Fahraeus and pinched flow fractionation effect	97	3.47	200	30
Sollier et al. [4]	Plasma Skimming	-	17.8	100	2
Kuo & Chen [29]	Centrifugation	-	96	-	6
Chen et al. [30]	Dielectrophoresis and Capillary Force	89.4	69.8	-	Whole blood
Dimov et al. [31]	Sedimentation	100	-	0.83	Whole blood
Aran et al. [32]	Cross Flow Filtration	100	15	10	30
Li et al. [33]	Dead End Filtration	100	2	0.02	Whole blood
Chung et al. [34]	Dead End Filtration	100	14	50	Whole blood
Mach & Di Carlo [35]	Inertial Force	100	-	8000	0.225
Lenshof et al. [36]	Acoustophoresis	100	-	80	40
Jiang et al. [37]	Dielectrophoresis	100	26.6	-	2.8
Madadi et al. [11]	Capillary force and filtration	>98	3.6	1–1.7	Whole blood
Current study	Zweifach-Fung	100	32	80	Whole blood

## Supplementary Materials

The supplementary materials are available online at http://www.mdpi.com/1424-8220/16/9/1543/s1.
