# Supplementary material for: Biomimetic Precapillary Flow Patterns for Enhancing Blood Plasma Separation: A Preliminary Study
Source: Sensors (Basel). 2016 Sep 21;16(9):1543. doi: 10.3390/s16091543 (PMC5038815; doi:10.3390/s16091543)
Supplement: Supplementary file 1 [file sensors-16-01543-s001.pdf]

Sensors **2016**, *16*, 1543; doi:10.3390/s16091543

Supplementary Materials: Biomimetic Precapillary Flow Patterns for Enhancing Blood Plasma Separation: A Preliminary Study

Bumseok Namgung, Justin Kok Soon Tan, Peter Agustinus Wong, Sung-Yong Park, Hwa Liang Leo and Sangho Kim

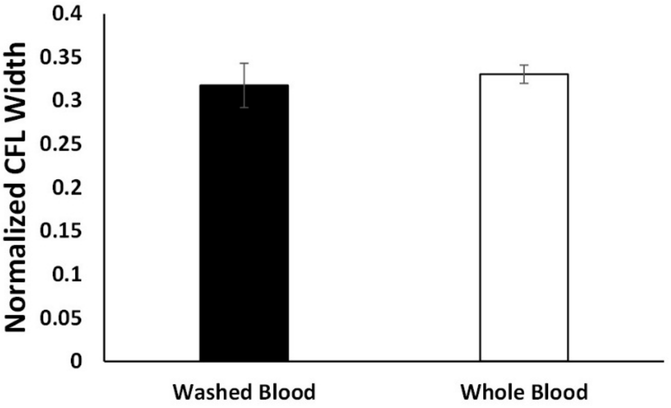

Figure S1. Comparison between the use of washed RBCs re-suspended in PBS and whole blood on the separation performance. Both samples were matched to 47.5% hematocrit. Statistical testing revealed no significant difference between the normalized CFL widths for both samples ($P > 0.05$).

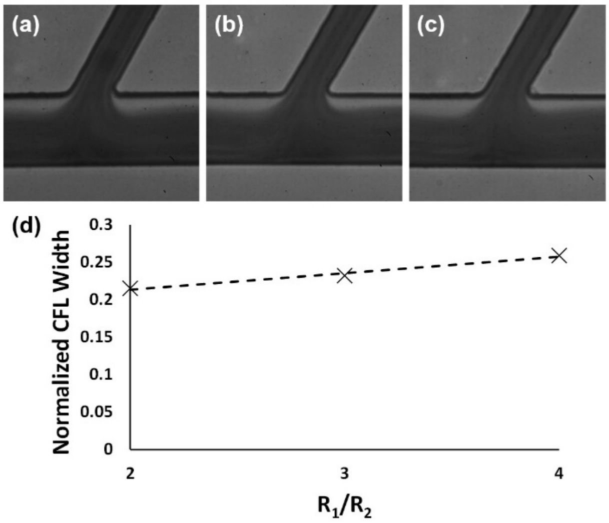

Figure S2. Typical examples for plasma separation from 40% hematocrit blood samples with the flow resistance differences of $R_1/R_2 = 2$ (**a**); $R_1/R_2 = 3$ (**b**) and $R_1/R_2 = 4$ (**c**); (d) Plot of normalized CFL width against R_1/R_2. The normalized CFL width increased linearly with R_1/R_2 due to increased flow biasing towards channel 2.
